# The Gap Junction Inhibitor Octanol Decreases Proliferation and Increases Glial Differentiation of Postnatal Neural Progenitor Cells

**DOI:** 10.3390/ijms25126288

**Published:** 2024-06-07

**Authors:** Rocío Talaverón, Camilo J. Morado-Díaz, Alejandro Herrera, Victoria Gálvez, Angel M. Pastor, Esperanza R. Matarredona

**Affiliations:** 1 Departamento de Bioquímica y Biología Molecular, Facultad de Farmacia, Universidad de Sevilla, 41012 Seville, Spain; rtalaveron@us.es; 2 Departamento de Fisiología, Facultad de Biología, Universidad de Sevilla, 41012 Seville, Spain; cmorado@us.es (C.J.M.-D.); ampastor@us.es (A.M.P.)

**Keywords:** connexin, subventricular zone, neural stem cells, proliferation, differentiation, glioblastoma

## Abstract

Neural precursor cells (NPCs) that persist in the postnatal/adult subventricular zone (SVZ) express connexins that form hemichannels and gap junctions. Gap junctional communication plays a role in NPC proliferation and differentiation during development, but its relevance on postnatal age remains to be elucidated. In this work we aimed to evaluate the effect of the blockade of gap junctional communication on proliferation and cell fate of NPCs obtained from the SVZ of postnatal rats. NPCs were isolated and expanded in culture as neurospheres. Electron microscopy revealed the existence of gap junctions among neurosphere cells. Treatment of cultures with octanol, a broad-spectrum gap junction blocker, or with Gap27, a specific blocker for gap junctions formed by connexin43, produced a significant decrease in bromodeoxyuridine incorporation. Octanol treatment also exerted a dose-dependent antiproliferative effect on glioblastoma cells. To analyze possible actions on NPC fate, cells were seeded in the absence of mitogens. Treatment with octanol led to an increase in the percentage of astrocytes and oligodendrocyte precursors, whereas the percentage of neurons remained unchanged. Gap27 treatment, in contrast, did not modify the differentiation pattern of SVZ NPCs. Our results indicate that general blockade of gap junctions with octanol induces significant effects on the behavior of postnatal SVZ NPCs, by reducing proliferation and promoting glial differentiation.

## 1. Introduction

Connexins are a family of proteins that form hemichannels and gap junctions. There are 21 different connexin isoforms that differ mainly in their molecular weight and C-terminal domain [1,2]. When present as hemichannels, connexins form undocked channels by which cells can contact the extracellular environment providing a pathway for the transfer of small ions or low molecular weight molecules between the cytosol and the extracellular space [3]. Hemichannels from two adjacent cells can dock to form gap junction channels. These gap junctions mediate the direct exchange of ions, second messengers, metabolites, and microRNAs between communicating cells which allows the synchronization of cell functions and the mediation of electrical and biochemical communication in a wide variety of cells [4,5]. In addition to these channel-dependent functions, the cytoplasmic domain of connexins can directly interact with other molecules to regulate intracellular signaling [6].

There are two brain regions in the postnatal and adult mammal brain in which neural progenitor cells (NPCs) persist: the dentate gyrus of the hippocampus and the subventricular zone (SVZ), lining the walls of the lateral ventricles. NPCs from the SVZ form neuroblasts that migrate towards the olfactory bulb where they generate new neurons [7,8]. NPCs of the SVZ can be isolated and amplified in vitro as floating cellular aggregates known as neurospheres [9,10,11]. NPCs from the postnatal and adult SVZ express connexin 43 (Cx43, molecular weight of about 43 kDa), Cx45, and Cx26 as well as pannexin1 [12,13]. In addition, neurosphere cells obtained from the postnatal SVZ are coupled by gap junctions [12], a situation also observed in NPCs in the postnatal SVZ neurogenic niche [14]. To date, some concrete actions of gap junctional communication in postnatal/adult SVZ-derived NPCs have been reported such as improvement of survival [15], maintenance of the cellular architecture of the SVZ niche [16], or direct communication with blood vessels [14]. In embryonic NPCs from the SVZ, gap junctions between NPCs have a role in the maintenance of a self-renewal state [17,18], an effect also reported for gap junctional communication in NPCs from other sources such as the ventral mesencephalon [19], the caudal neural tube of E10.5 embryos [20], or the inner cell mass of human [21] or mouse blastocysts [22]. However, putative effects of gap junctional communication in the control of postnatal SVZ proliferation have not been reported so far.

Several lines of evidence suggest that some types of glioblastomas (GBM) originate from SVZ NPCs, upon acquisition of driver mutations [23,24]. GBM is the most common and aggressive primary brain tumor [25]. Despite advances in basic and clinical research, this cancer remains incurable so there is an urgent need of new therapies. The knowledge of factors controlling NPC proliferation in the SVZ may constitute a valuable tool in the search for putative alternative therapies for this cancer, aimed to target the cell of origin.

Gap junction-mediated intercellular communication is also required for differentiation during brain development. For instance, in embryonic NPCs, uncoupling of cells via pharmacological blockade of gap junctions or via down-regulation of Cx43 induces a decrease in the acquisition of the neuronal phenotype [19,26]; this suggests that gap junctional communication is necessary to complete neuronal differentiation. In addition, mechanisms that include differential expression of connexins are involved in astrocytic and neuronal commitment during development since connexin expression by NPCs dynamically changes as the central nervous system matures [27,28,29,30]. In adult neurogenesis, mice lacking Cx30 and Cx43 show a significant decline in NPC proliferation and in the formation of new neurons in the hippocampal dentate gyrus [31,32]. Therefore, while it has been demonstrated that intercellular communication via gap junctions is important for NPC proliferation and differentiation during development and in adult hippocampal neurogenesis, the relevance of such type of communication in NPC proliferation and differentiation of the SVZ during the postnatal age has not been explored.

In this article we aimed to evaluate the effects of the blockade of gap junctions on the proliferation and differentiation of NPCs, isolated from the SVZ of postnatal rats, using a broad-spectrum gap junction blocker, octanol, and a Cx43 gap junction blocker, Gap27. Based on similarities between SVZ NPCs and glioma stem cells, we also evaluated the effect of octanol on the growth of two glioma cell lines derived from patients. Our results showed that NPC proliferation in neurospheres was diminished after treatment with the gap junction inhibitors octanol and Gap27. The growth of glioma stem cells was also reduced by octanol treatment. In addition, we demonstrated that octanol, but not Gap27 treatment, favored NPC differentiation towards glial phenotypes.

## 2. Results

**Figure 1:**
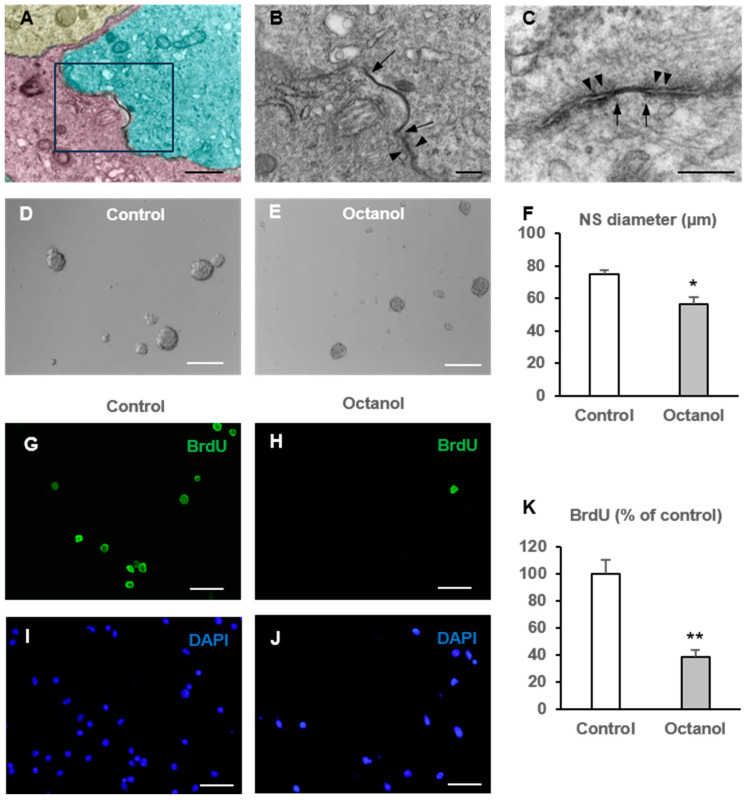
Effect of octanol treatment on neural progenitor cell proliferation. (**A**) Electron microscopy image of cells within a neurosphere. The different identified cells have been pseudocolored. The framed region shows an example of a zone of gap junction-like contact between two apposed cells. (**B**) Shows the framed region of (**A**) at higher magnification. (**C**) Shows another example of a gap junction-like contact between two intimately apposed neurosphere cells. Note the limits of gap junction-like contacts (delimited by arrows) in the two cell membranes and adjacent desmosomal-like junctions (arrowheads). Scale bars: 20 µm in (**A**), and 5 µm in (**B**,**C**). Neural progenitor cells of the postnatal rat subventricular zone were cultured in the absence (control) or presence of the gap junction inhibitor octanol. The diameter of the formed neurospheres 72 h after seeding, as well as the percentage of bromodeoxyuridine (BrdU) incorporation during the last 12 h of culture were evaluated. (**D**,**E**) Phase-contrast photomicrographs of neurospheres in control cultures (**D**) and in cultures treated with 0.5 mM octanol (**E**). Scale bar: 100 µm. (**F**) Graph showing the neurosphere (NS) diameter (in µm) in each experimental condition. Data are the mean ± SEM, n = 5, * *p* < 0.05, Student’s *t* test. (**G**–**J**) Epifluorescence images showing BrdU immunohistochemistry (in green) in cells from gently disaggregated neurospheres obtained from a control culture (**G**) and a culture treated with 0.5 mM octanol (**H**). The total number of cells in each field was identified via DAPI staining (in blue, (**I**,**J**)). Scale bar: 50 µm. (**K**) Graph showing the percentage of BrdU incorporation in both experimental conditions. Data are the mean ± SEM, n = 8, ** *p* < 0.001, Student’s *t* test.

We have previously shown that NPCs of the postnatal SVZ express connexins and pannexins when they are isolated and grown as neurospheres [12,33]. Now we showed via electron microscopy the existence of gap junctions among SVZ-derived neurosphere cells. As seen in Figure 1A–C, gap junction-like contacts were frequently identified at the electron microscopy level between adjacent neurosphere cells (see arrows). Some of the identified gap junction-like contacts were delimited by desmosomal-like junctions, characterized by the presence of intermediate filaments protruding perpendicular to the junction (Figure 1B,C; arrowheads).

Next, we wanted to explore whether gap junctional communication blockade in neurospheres affected cell proliferation. For that purpose, we used a broad-spectrum gap junction blocker, octanol, that blocks gap junctions with little selectivity for the connexin isoform that constitutes the apposed hemichannels [34]. This inhibitory action has been associated to deleterious effects on membrane fluidity (reviewed in [35]). We performed our experiments with octanol 0.5 mM, since we have previously demonstrated that this concentration of octanol effectively inhibits gap junctions in SVZ-derived neurospheres [12]. Neurospheres were grown in parallel in control conditions (cultures treated with the vehicle) and in octanol-treated conditions (cultures treated with octanol 0.5 mM). After 72 h of culture, images of neurospheres were taken and their diameter analyzed. The diameter of the neurospheres grown in the presence of octanol 0.5 mM was reduced by 25% as compared to the diameter of control neurospheres (Figure 1D–F) (Student’s *t* test; n = 5, *p* = 0.004). To analyze whether the decrease in the neurosphere diameter was due to a lower proliferation rate, we aimed to analyze bromodeoxyuridine (BrdU) incorporation in neurospheres after treatment with octanol during the time of culture. For that purpose, we added the timidine analogue BrdU for the last 12 h of culture. Collected neurospheres from control and treated cultures were gently dissociated, placed on coverslips, and processed for BrdU immunohistochemistry. Treatment of neurospheres with 0.5 mM octanol significantly reduced BrdU incorporation in the cells to a 38.6 ± 4.8% with respect to control cultures (Figure 1G–K) (Student’s *t* test, n = 8, *p* < 0.001). As neurosphere size might also be reduced by cell death, we evaluated the degree of apoptotic death in NPC cultures in the absence and presence of 0.5 mM octanol, via the terminal dUTP nick end labeling (TUNEL) assay. The percentage of apoptotic cells did not significantly differ between control and octanol-treated cultures (Figure S1). Therefore, we concluded that the decrease in neurosphere size observed in octanol-treated neurospheres was indeed due to a decrease in cell proliferation. We also analyzed the number of neurospheres formed in each experimental condition. Octanol did not produce a significant effect on the number of neurospheres formed after 72 h of treatment (13.5 ± 2.6 vs. 12.5 ± 1.4 neurospheres/mm^2^ for control and octanol treated cultures, respectively, *p* = 0.54, Student’s *t* test, n = 5). This indicated that the self-renewal activity of postnatal SVZ-derived NPCs was not affected by octanol treatment.

**Figure 2:**
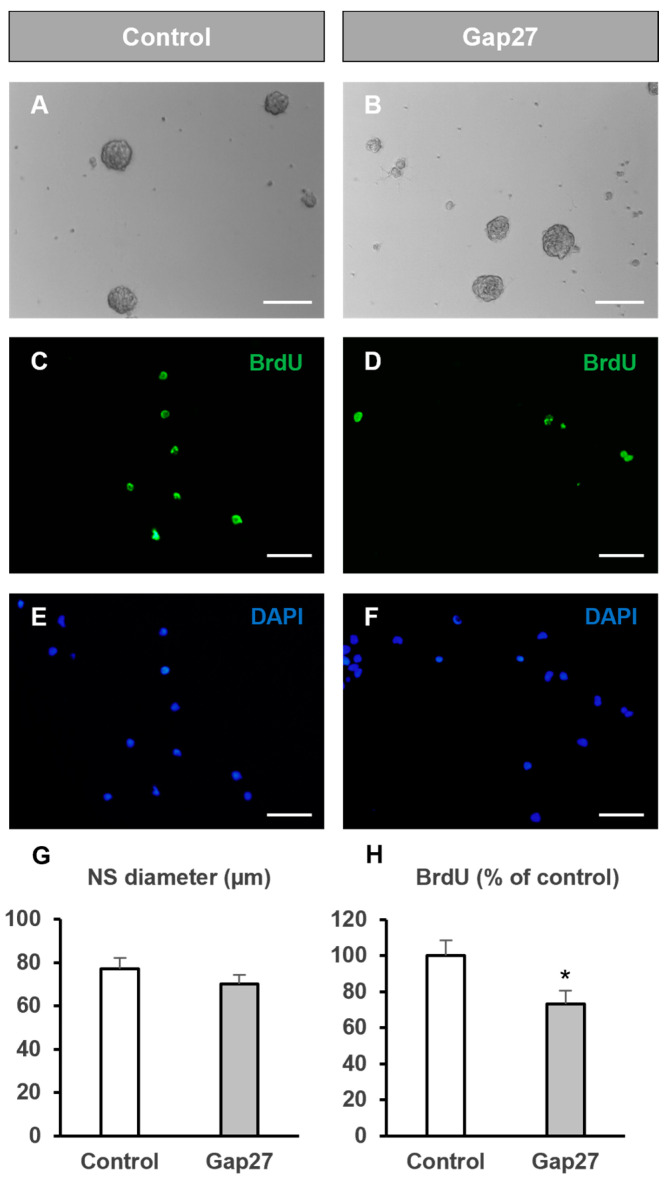
Effect of Gap27 treatment on neural progenitor cell proliferation. Neural progenitor cells of the postnatal rat subventricular zone were cultured in the absence (control) or presence of the Cx43 gap junction inhibitor peptide Gap27. The diameter of the formed neurospheres 72 h after seeding as well as the percentage of bromodeoxyuridine (BrdU) incorporation during the last 12 h of culture were evaluated. (**A**,**B**) Phase-contrast photomicrographs of neurospheres in control cultures (**A**) and in cultures treated with 0.3 mM Gap27 (**B**). Scale bar: 100 µm. (**C**–**F**) Epifluorescence images showing BrdU immunohistochemistry (in green) in neurosphere-derived cells obtained from control cultures (**C**) and cultures treated with 0.3 mM Gap27 (**D**). The total number of cells in each field was identified via DAPI staining (in blue, (**E**,**F**)). Scale bar: 50 µm. (**G**) Graph showing the neurosphere (NS) diameter (in µm) in each experimental condition. Data are the mean ± SEM, n = 6, *p* > 0.05, Student’s *t* test. (**H**) Graph showing the percentage of BrdU incorporation in both experimental conditions. Data are the mean ± SEM, n = 8, * *p* < 0.05 Student’s *t* test.

We repeated the experiments using Gap27, a synthetic Cx43 mimetic peptide that, in the range of 100 to 300 µM, selectively blocks gap junctions formed by Cx43 [36,37]. Gap27 interacts with an amino acid sequence of the second extracellular loop of Cx43 hemichannels, preventing their docking and hence the formation of Cx43 gap junctions [38]. NPC treatment with 300 µM of Gap 27 did not significantly modify neurosphere size compared to controls (Figure 2A,B,G) (Student’s *t* test; n = 6, *p* = 0.17) but led to a significant reduction in BrdU incorporation (73.3 ± 7.7% compared to control) (Figure 2C–F,H) (Student’s *t* test, n = 8, *p* = 0.043). Therefore, specific blockade of Cx43-forming gap junctions also diminished the proliferation rate in SVZ neurospheres, although in a significantly lower percentage than that induced via octanol (73.3 ± 7.7% for Gap27 vs. 38.6 ± 4.8% for octanol, *p* = 0.007, ANOVA). NPC self-renewal was not affected by Gap 27 treatment since the number of neurospheres formed after 72 h of treatment did not significantly differ with respect to control cultures (11.4 ± 1.3 vs. 13.0 ± 1.2 neurospheres/mm^2^ for control and treated cultures, respectively, *p* > 0.05, Student’s *t* test, n = 6).

**Figure 3:**
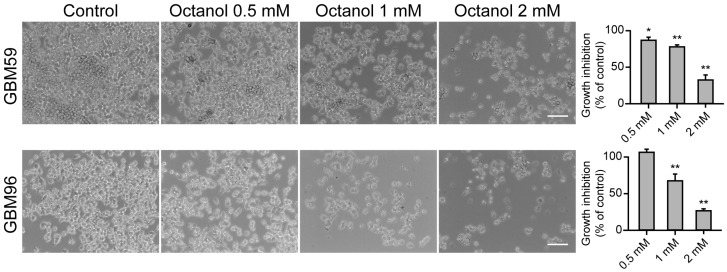
Effect of octanol on glioblastoma cell growth. Cell viability in GBM cells treated for 72 h with different concentrations of octanol (0.5, 1 and 2 mM). Phase-contrast photomicrographs in the left panel show examples of GBM59 and GBM96 cells in control conditions and after 72 h of treatment with octanol at the indicated concentrations. Scale bars: 100 µm. Bar histograms in the right panel show the growth inhibition (as the percentage with respect to control) of every tested concentration of octanol in GBM59 and in GBM96 cells. Data are the mean ± SEM, n = 6–12, * *p* < 0.05, ** *p* < 0.001, compared to control, one-way ANOVA test.

SVZ NPCs share common features with glioma stem cells and have been proposed as cells of origin of GBM upon acquisition of driver mutations [23,24]. Based on this background, and in the obtained results with octanol in SVZ NPCs, we decided to explore possible antiproliferative actions of octanol on primary human GBM cells. For that purpose, we used the MTT assay and evaluated cell growth with different concentrations of octanol. As seen in Figure 3, octanol induced a dose-dependent reduction in cell growth in the two tested GBM cell lines, GBM 59 and GBM96. Higher concentrations of octanol were required to exert similar reductions in cell growth to that obtained in SVZ NPCs, which indicated that these tested tumoral cells were less sensitive to octanol antimitotic action than SVZ NPCs.

**Figure 4:**
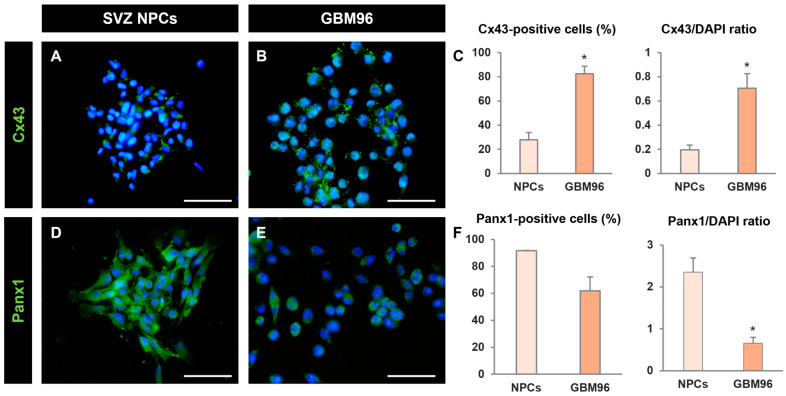
Comparison of hemichannel protein expression between neural progenitor cells and glioblastoma cells. (**A**,**B**,**D**,**E**) Epifluorescence microscopy images of SVZ NPCs and GBM96 cells after immunostaining for the detection of Cx43 (green, (**A**,**B**)) or Panx1 (green, (**D**,**E**)). Cell nuclei are identified via staining with DAPI (in blue). Scale bars: 50 µm. (**C**) Graphs showing the percentage of Cx43-positive cells and the Cx43/DAPI ratio in SVZ NPCs (NPCs) and in GBM96 cells. Data are the mean ± SEM, n = 5, * *p* < 0.05, GBM96 compared to NPCs, Student’s *t* test. (**F**) Graphs showing the percentage of Panx1-positive cells and the Panx1/DAPI ratio in SVZ NPCs (NPCs) and in GBM96 cells. Data are the mean ± SEM, n = 3–5, * *p* < 0.05, GBM96 compared to NPCs, Student’s *t* test.

Next, we analyzed possible differences in hemichannel protein expression between SVZ NPCs and GBM cells. Both the percentage of Cx43-positive cells and the Cx43/DAPI ratio were significantly higher in GBM96 cells than in SVZ NPCs (Figure 4A–C). Conversely, Panx1 expression was more robust in SVZ NPCs compared to GBM96 cells, with nearly 100% of NPCs expressing this protein and showing an intense labeling (Figure 4D–F).

**Figure 5:**
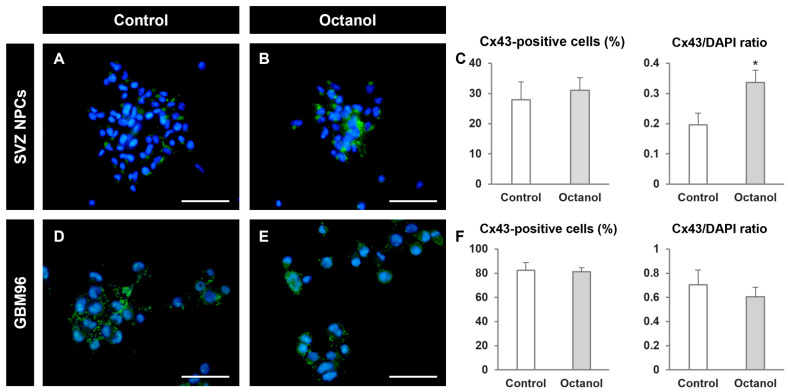
Octanol treatment increased Cx43 expression in SVZ NPCs. (**A**,**B**,**D**,**E**) Epifluorescence microscopy images of SVZ NPCs and GBM96 cells in control (**A**,**D**) and in octanol-treated conditions (**B**,**E**) after immunostaining for the detection of Cx43 (green). Cell nuclei are identified by staining with DAPI (in blue). Scale bars: 50 µm. (**C**,**F**) Graphs showing the percentage of Cx43-positive cells and the Cx43/DAPI ratio in SVZ NPCs (**C**) and in GBM96 cells (**F**), in control conditions and after treatment with octanol 0.5 mM (for NPCs) or 1 mM (for GBM96 cells). Data are the mean ± SEM, n = 5–6, * *p* < 0.05, GBM96 compared to NPCs, Student’s *t* test.

Octanol treatment did not induce significant modifications in the percentage of Cx43-positive cells either in SVZ NPCs nor in GBM96 cells (Figure 5A–F). However, the ratio Cx43/DAPI was significantly increased by octanol treatment in SVZ NPCs (Figure 5A–C). This indicated that Cx43-positive NPCs responded to octanol treatment with an increased expression of the protein. The effect of octanol on Cx43 expression was not observed in GBM96 cells (Figure 5D–F).

Neither the percentage of Panx1-positive cells or the Panx1/DAPI ratio were significantly modified by treatment of SVZ NPCs or GBM96 cells with octanol (Table S1).

**Figure 6:**
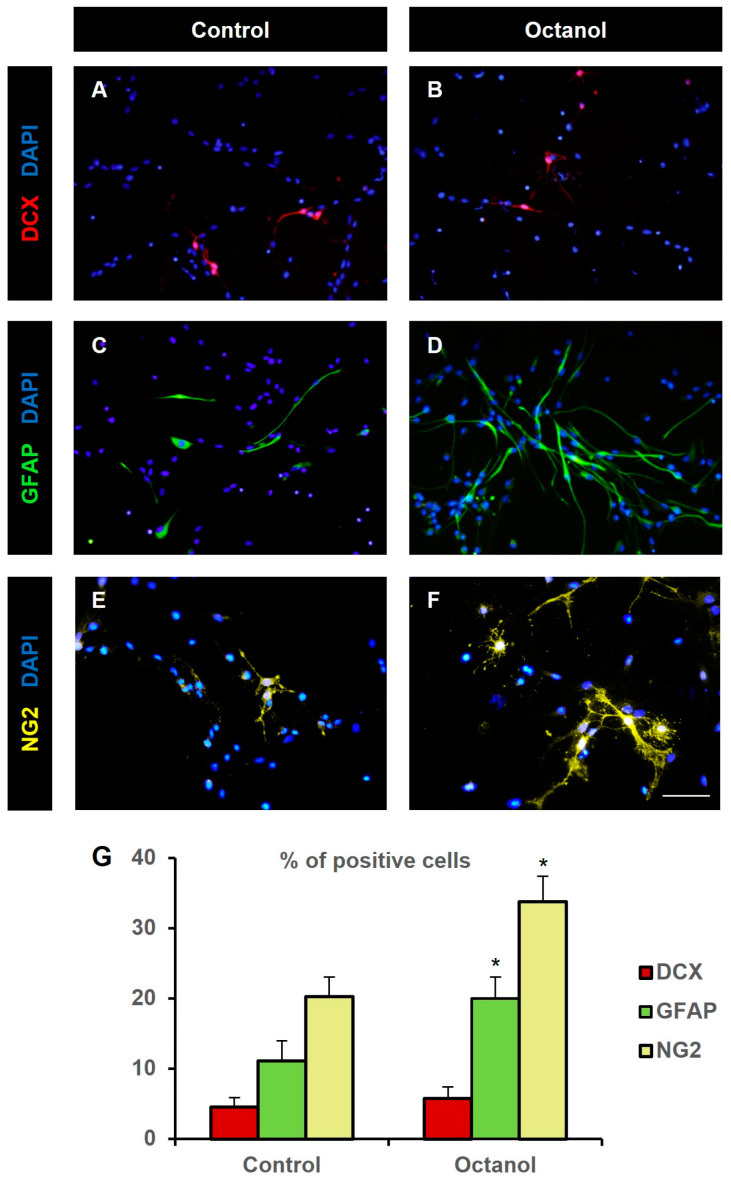
Octanol treatment increased glial differentiation in vitro. Fluorescence microscopy images of neurosphere-derived adhered cells immunostained with antibodies to identify neurons (DCX, red, (**A**,**B**)), astrocytes (GFAP, green, (**C**,**D**)) and oligodendroglial precursors (NG2, pseudocolored in yellow, (**E**,**F**)) and counterstained with DAPI (blue), in cultures treated for 48 h with vehicle (control, (**A**,**C**,**E**)) or 0.5 mM octanol (**B**,**D**,**F**). Note a higher percentage of GFAP- and NG2-positive cells in octanol-treated cultures compared to control ones. Scale bar: 50 µm. (**G**) Percentage of cells immunoreactive to DCX (red), GFAP (green), and NG2 (yellow) in neurosphere-derived cells grown on adhesive substrate for 48 h and treated either with vehicle (control) or with 0.5 mM octanol. Data are the mean ± SEM of 7 to 8 independent experiments. * *p* < 0.05 compared to their respective control (Mann–Whitney U test for DCX and GFAP data, Student’s *t* test for NG2 data).

Some studies have reported that gap junction communication intervenes in determining cell fate during development [19,26]. Therefore, our next goal was to evaluate the effect of octanol on SVZ NPC differentiation. For that purpose, neurosphere-derived NPCs were mechanically dissociated and seeded on an adhesive substrate in the absence of EGF and FGF-2. Octanol 0.5 mM was added to the medium and cultures were maintained for 48 h after which the differentiation to neurons, astrocytes, and oligodendrocyte precursors was evaluated. We observed that differentiation towards neurons was not affected by the treatment (4.5 ± 1.4% in control cultures vs. 5.8 ± 1.6% in cultures treated with octanol (Mann–Whitney U test, n = 7, *p* = 0.54; Figure 6A–G). However, octanol treatment promoted NPC differentiation to astrocytes and to oligodendrocyte progenitors, as demonstrated by the significant increase in the percentage of GFAP-immunoreactive cells (11.1 ± 2.9% in control cultures vs. 20.0 ± 3% in cultures treated with octanol, Mann–Whitney U test, n = 8, *p* = 0.034; Figure 6C,D,G) and in the NG2-immunoreactive cells (20.2 ± 2.8% in control cultures vs. 33.7 ± 3.6% in cultures treated with octanol, Student’s *t* test, n = 7, *p* = 0.009; Figure 6E–G).

**Figure 7:**
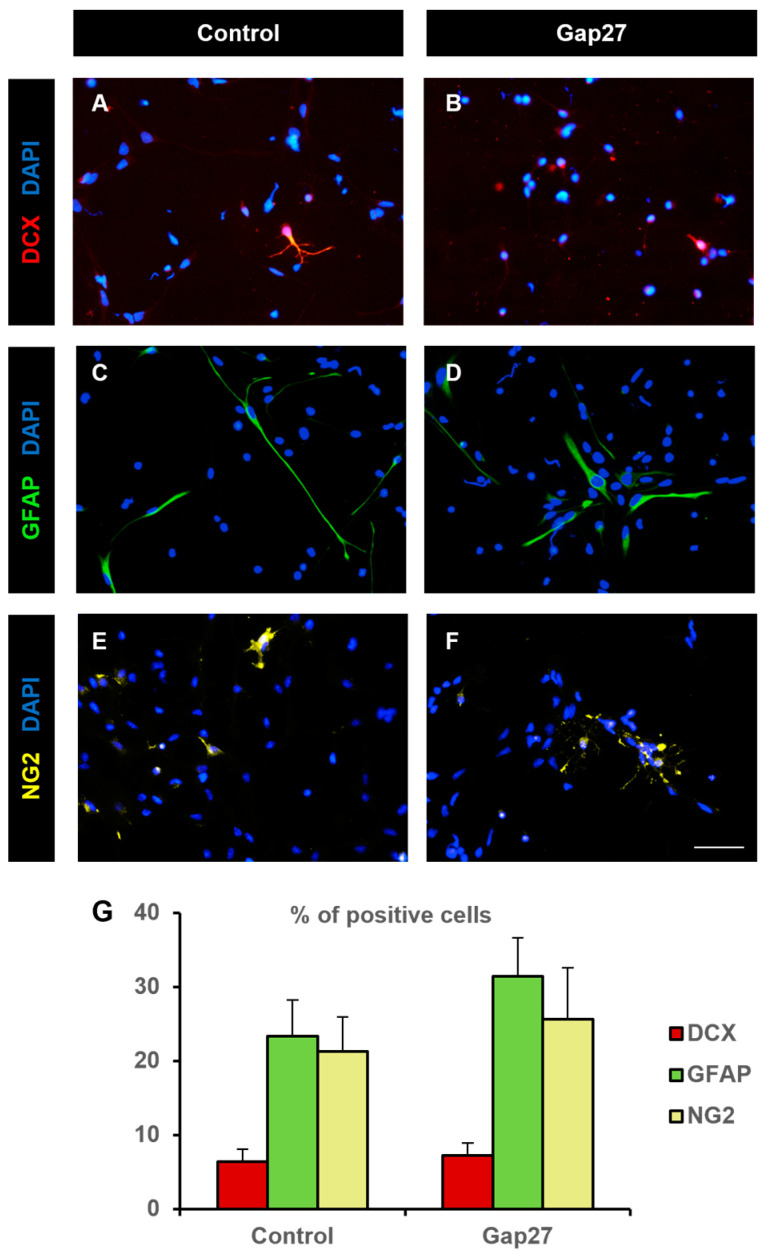
Neural progenitor cell fate was not affected by Gap27 treatment. Fluorescence microscopy images of neurosphere-derived adhered cells immunostained with antibodies to identify neurons (DCX, red, (**A**,**B**)), astrocytes (GFAP, green, (**C**,**D**)), and oligodendroglial precursors (NG2, pseudocolored in yellow, (**E**,**F**)) and counterstained with DAPI (blue), in cultures treated for 48 h with vehicle (control, (**A**,**C**,**E**)) or 0.3 mM Gap27 (**B**,**D**,**F**). Scale bar: 50 µm. (**G**) Percentage of cells immunoreactive to DCX (red), GFAP (green), and NG2 (yellow) in neurosphere-derived cells grown on adhesive substrate for 48 h and treated either with vehicle (control) or with 0.3 mM Gap27. Data are the mean ± SEM of five independent experiments, no significant differences were found in the comparisons between Gap27 treatment values and their respective controls (Mann–Whitney U test for GFAP data, Student’s *t* test for DCX and NG2 data).

The effect of the Cx43 gap junction-specific blocker Gap27 on cell fate was also analyzed. Treatment of NPCs with 0.3 mM Gap27 cultured on adhesive substrate in the absence of mitogens did not induce any significant modification in the percentage of cells that underwent differentiation towards neurons, astrocytes, or oligodendrocyte precursors (Figure 7). The percentage of NPCs that differentiated to neurons, astrocytes, and oligodendrocyte precursors in control cultures was 6.4 ± 1.7%, 23.3 ± 4.9%, and 21.6 ± 4.6%, respectively (Figure 7A,C,E,G). Similar percentages were obtained in cultures treated with Gap27: 7.2 ± 1.7% neurons, 31.4 ± 5.2% astrocytes, and 25.6 ± 7.0% oligodendrocyte precursors (Figure 7B,D,F,G). Comparisons of these values with their controls revealed no significant differences (Mann–Whitney U test for GFAP data, Student’s *t* test for DCX and NG2 data, *p* > 0.05).

## 3. Discussion

The intercellular passage of ions and molecules among coupled cells by gap junctions facilitates the coordination and synchronization of various processes that affect important cellular functions such as growth, differentiation, survival, and death [39,40,41]. Specifically, during brain development, NPCs communication by gap junctions has been reported to be required for maintaining NPCs in a proliferative state [17,18,22]. Also in the adult brain, gap junctions between NPCs are necessary to maintain hippocampal neurogenesis [31] and to achieve an adequate integration in the hippocampal circuitry of newly generated dentate granule cells [42]. In our study, we showed via electron microscopy that NPCs from the postnatal SVZ formed gap junction-like contacts when they were grown in vitro as neurospheres. This result was in accordance with our previous report showing dye coupling among cells in SVZ neurospheres and between neurosphere cells and glial cultures [12,33]. We also showed that gap junctional communication among neurosphere cells intervened in the control of NPC proliferation since both its general blockade with octanol and with a Cx43-forming gap junction blocker (Gap27) reduced BrdU incorporation. In vivo, evidence of gap junctional communication among NPCs and between NPCs and astrocytes in the postnatal SVZ has been reported [14]. In this mentioned work, the authors also demonstrated the propagation of calcium waves through these gap junctions. In relation to this, proliferation of postnatal SVZ NPCs correlated with an increase in blood flow which is dependent on calcium [43]. Based on this evidence and on our results in vitro, we hypothesize that gap junctions might intervene in the control of NPC proliferation in the postnatal SVZ neurogenic niche.

It is important to highlight that octanol exerts a more robust antiproliferative action in NPCs than Gap27. Several explanations may account for this result. First, octanol produces a general blockade of gap junctions whereas Gap27 only those formed by Cx43. Second, octanol also produces a general blockade of hemichannels besides gap junctions [44]. We have recently reported that the blockade of hemichannels with boldine significantly reduces the proliferation of postnatal SVZ NPCs [13]. Third, octanol treatment leads to an increase in Cx43 expression in NPCs. In a previous study, we showed that Cx43, specifically the region 266–283 of its intracellular C terminus domain, inhibits the activity of the nonreceptor tyrosine kinase Src in SVZ NPCs, and reduces their proliferation [45]. These results indicate that an increase in Cx43 expression may also affect NPC proliferation through channel-independent mechanisms. Besides all this, other channels and receptors such as T-type calcium channel or P2X7 receptors have been shown to be inhibited by octanol [44,46]. This implies that we cannot certainly state that the antiproliferative action of octanol in SVZ NPCs is exclusively mediated by the inhibition of gap junctions. Additional mechanisms of actions such as hemichannel blockade, inhibition of T-type calcium channel and other channels or receptors, or effects derived from the increased expression of Cx43 may be involved in the antimitotic action.

Glioma stem cells have many similarities with SVZ NPCs, such as nestin expression, high motility, diversity of progeny, proliferative potential, association with blood vessels, and bilateral communication with constituents of the niche [47]. Our results showed that octanol also induced a dose-dependent antimitotic action in patient-derived glioblastoma cells. However, higher concentrations were required to achieve similar antimitotic actions than with NPCs which indicated that these types of tumoral cells were less sensitive than SVZ NPCs to octanol treatment. Again, different mechanisms of action, not only gap junctional inhibition, may account for these results, based on the wide variety of channels and receptors that are inhibited by octanol treatment. For instance, drugs the specifically block hemichannels (e.g., boldine), gap junctions (e.g., carbenoxolone), or T-type calcium channels (e.g., mibefradil) have been shown to inhibit the proliferation of GBM cells [13,48,49]. Further experiments will have to be carried out to unravel the precise mechanism of action of the antiproliferative effect of octanol in GBM cells.

NPCs from SVZ neurospheres generate neurons, astrocytes, and oligodendrocytes when they are induced to differentiate [50,51]. Many molecules and pathways have been described to be involved in the switch from proliferation to differentiation in NPCs but, to date, the importance of gap junctional-mediated communication during this state transition has not been evaluated in detail. We showed that octanol treatment of neurosphere-derived NPCs in the absence of mitogens induced a significant increase in the percentage of astrocytes and oligodendrocyte precursors. According to our results, differentiation towards glial phenotypes may be restricted while cells were coupled by gap junctional communication. Alternatively, other mechanisms, besides gap junctional inhibition, may explain these results obtained via octanol treatment. For instance, molecules released by hemichannels could intervene in NPC determination. Candidates for hemichannel-passing molecules are glutamate or purines such as ATP. Inhibition of glutamate release via hemichannel blockade has been reported to induce the differentiation of oligodendrocyte precursors to mature oligodendrocytes in hypoxic conditions [52]. With respect to ATP, NPCs of the SVZ express different types of purinergic receptors so they can respond to ATP released by hemichannels [53] and modify their behavior. Specifically, the effects of ATP signaling on SVZ NPC differentiation remain to be elucidated but, according to our results with the general gap junction and hemichannel blocker octanol, a role of ATP in the restriction to the glial phenotype of this population of progenitor cells cannot be ruled out.

We have demonstrated that communication mediated by gap junctions formed by Cx43 probably do not intervene in the control of NPC fate since treatment with Gap27 did not modify the differentiation pattern of SVZ NPCs. This result agreed with previous studies performed in Cx43 null mice in which the lack of Cx43 altered calcium signaling and affected NPC proliferation and migration but not NPC differentiation during development [54]. As mentioned earlier, connexins may have other roles in NPC proliferation and differentiation not dependent on their function as channels. Santiago et al. (2010) [55] showed that the carboxyl-terminal domain of Cx43 prevents premature neuronal differentiation during embryonic brain development. A cross-regulation of Cx43 and β-catenin has also been reported with relevance in the induction of neuronal differentiation [56]. Recently, we have demonstrated that the region 266–283 of the intracellular domain of Cx43 increases astrocytic differentiation in SVZ NPCs via mechanisms involving the down regulation of β-catenin [45].

To sum up, our results with neurospheres formed by NPCs obtained from the postnatal SVZ showed that treatment with the gap junction blockers octanol and Gap27 induced a decrease in NPC proliferative activity, being more potent with octanol than with Gap27. Treatment with octanol also reduced cell growth of GBM cells. In differentiation-inducing conditions, culture treatment with octanol favored NPC differentiation towards the glial lineage. These results are indicative of important functions of communication routes mediated by connexin channels in the control of proliferation and differentiation of SVZ postnatal NPCs.

## 4. Materials and Methods

### 4.1. Neural Precursor Cell Culture

Experiments were carried out with 7-day postnatal Wistar rats (P7) (*Rattus norvegicus*) of either sex according to the guidelines of the European Union (2010/63/EU) and Spanish law (R.D. 53/2013 BOE 102 34/11370-420, 2013) for the use and care of laboratory animals. Experimental procedures used in this study were approved by the ethics committee of the Universidad de Sevilla.

NPCs were isolated from the SVZ of P7 rats and were expanded in the form of neurospheres. Four P7 rats were used for every independent culture. Briefly, the lateral walls of the lateral ventricles were removed and enzymatically dissociated with 1 mg/mL trypsin (Invitrogen, Thermo Fisher Scientific, Waltham, MA, USA) at 37 °C for 15 min. The tissue was then centrifuged at 150× *g* for 5 min, rinsed in Dulbecco’s modified Eagle’s medium/F12 medium 1:1 (DF-12; Invitrogen), and centrifuged again in the same conditions. Then, the cells were resuspended in DF-12 containing 0.7 mg/mL ovomucoid (Sigma-Aldrich, St. Louis, MO, USA), and mechanically disaggregated with a fire-polished Pasteur pipette. The dissociated cells were centrifuged and resuspended in defined medium (DM: DF-12 containing B-27 supplement, 2 mM Glutamax^®^, 100 units/mL penicillin, 100 µg/mL streptomycin and 0.25 µg/mL amphotericin B, all from Invitrogen) supplemented with 20 ng/mL epidermal growth factor (EGF; PeproTech, Rocky Hill, NJ, USA), and 10 ng/mL basic fibroblast growth factor (FGF-2; Millipore, Temecula, CA, USA). The cell suspension was maintained in an atmosphere of 5% CO_2_, at 37 °C. After 1–2 days, cell aggregates named neurospheres were formed. Neurosphere cells were subcultured every 3–4 days via centrifugation, mechanical dissociation, and resuspension with fresh medium. All the experiments were performed with cells between passages 2 and 6.

### 4.2. Fixation and Neurosphere Preparation for Electron Microscopy

Neurospheres were centrifuged at 150× *g* for 5 min and the pellet was fixed with 2% of glutaraldehyde in 0.1 M phosphate buffer. After postfixation with 1% osmium tetroxide for 1 h, the pellet was stained in bloc with uranyl acetate, dehydrated, and included in Durcupan ACM resin (Fluka Chemie GmbH, Buchs, Switzerland). Ultrathin sections (60–80 nm) were obtained (Leica EM UC7) and were examined on a transmission electron microscope (Libra 120, Zeiss, Carl Zeiss Microscopy, Oberkochen, Germany).

### 4.3. Glioblastoma Cell Culture

Patient-derived primary GBM cells (GBM59 and GBM96 cells; kindly donated by Dr. Manuel Sarmiento, University of Seville) were grown and maintained in DF-12 supplemented with 10% fetal bovine serum (Invitrogen), 2 mM Glutamax^®^, 100 units/mL penicillin, 100 μg/mL streptomycin, and 0.25 μg/mL amphotericin B, all from Invitrogen.

### 4.4. Analysis of Proliferation in Neurospheres

Proliferation was evaluated by measuring neurosphere diameter, neurosphere number and bromodeoxyuridine (BrdU) incorporation.

Every experiment was performed in parallel with two T25 flasks seeded with neurosphere-derived cells from the same culture, at a density of 10,000 viable cells/cm^2^. One of the flasks received drug treatment, and the other flask (control) was treated with the corresponding vehicle. Drugs used in our study were: octanol (broad spectrum gap junction blocker, Sigma-Aldrich), and Gap27 (inhibitor of gap junctions formed by Cx43, Sigma-Aldrich).

Flasks treated with octanol were added with a volume from a 5% stock solution of octanol (dissolved in ethanol) to reach a final concentration of 0.5 mM or 1 mM. Control flasks were added with the same volume of the vehicle, ethanol. Due to their volatility, octanol and ethanol were added every 24 h to the cultures.

Treatment with Gap27 consisted of adding the drug at the time of seeding, from a 100X stock solution prepared in MilliQ^®^ water to reach final concentration of 0.3 mM. Control flasks were added with the same volume of MilliQ^®^ water.

Seventy-two hours after seeding, photographs of the neurospheres formed in the two flasks of every experiment (control and drug treatment) were captured using a Leica EC3 camera coupled to a phase-contrast microscope (Leica DMIL-LED) with a 10X objective (ten photographs of random fields per flask). The diameter and the number of neurospheres were measured with ImageJ (NIH). In every experiment, a mean value of neurosphere diameter and neurosphere number was obtained for each condition. Data are presented as the mean ± SEM of values obtained in five to seven experiments.

For analyzing BrdU incorporation, experiments were performed as described above with the novelty that BrdU (Sigma Aldrich; 1 µM) was added to the flasks for the last 12 h of culture. Then, collected neurospheres from each flask (control and drug treatment) were slightly disaggregated with a P200 pipette and seeded on 12 mm diameter coverslips with DF-12 containing 1% fetal calf serum (Invitrogen), to allow adhesion. After 1 h of seeding on the coverslips, cells were fixed with 4% paraformaldehyde in 0.1 M phosphate buffer (10 min incubation) and processed for BrdU immunohistochemistry.

### 4.5. BrdU Immunohistochemistry

Cells in the coverslips were exposed to a DNA denaturation treatment consisting of a 20 min incubation with 2N HCl, after several washes with phosphate-buffered saline (PBS). Subsequently, coverslips were immersed in borate buffer pH 8.5 for 10 min and then washed three additional times with PBS. Then, coverslips were incubated with a solution containing a BrdU antibody (see Table 1) for 2.5 h, prepared in 2.5% bovine serum albumin (Sigma-Aldrich) in PBS. After rinsing, coverslips were incubated for 30 min with anti-mouse IgG coupled to FITC (Jackson ImmunoResearch, West Grove, PA, USA, 1:200), washed several times with PBS, and counterstained with 4′-6′-diamidino-2-phenylindole (DAPI, Sigma-Aldrich, 0.1 µg/mL) for 10 min. After final washes, coverslips were mounted on slides with a n-propyl-gallate solution (Sigma-Aldrich, 0.1 M) prepared in glycerol:PBS 9:1.

### 4.6. Evaluation of Apoptosis

To analyze cell death via apoptosis, collected neurospheres from control and treated flasks were disaggregated and seeded on poly-L-ornithine-treated 12-mm diameter coverslips with DF-12 containing 1% fetal calf serum. After 1 h of seeding, coverslips were fixed for 15 min with 4% paraformaldehyde in 0.1 M phosphate buffer and processed for terminal dUTP nick end labeling (TUNEL) assay using the instructions provided in the kit (Molecular Probes, Click-iT^®^ Plus TUNEL Assay C10618).

### 4.7. MTT Assay

3-(4,5-dimethylthiazol-2-yl)-2,5-diphenyltetrazolium bromide (MTT) (Sigma-Aldrich) was used to determine the cell viability and the 50% growth inhibitory concentration (IC50) of octanol in GBM59 and in GBM96 cells. GBM cells were cultured in 12-well plates at a density of 5000 cells/well in GBM medium. Subsequently, octanol was added to the culture medium from a 5% stock prepared in ethanol to reach final concentrations of 0.5, 1, or 2 mM. Control wells received the same volume of the vehicle (ethanol). Octanol or ethanol were added every 24 h. After 72 h of culture, GBM cells were incubated in the dark with culture medium containing 0.5 mg/mL MTT. After 10 min in dimethyl sulfoxide (DMSO), absorbance was measured at a wavelength of 570 nm using a microplate reader (Thermo Scientific Multiskan GO, Thermo Fisher Scientific, Waltham, MA, USA.

### 4.8. Identification of Hemichannel Proteins via Immunocytochemistry

Neurospheres collected from control and octanol (0.5 mM) treated flasks were slightly disaggregated and seeded on 12 mm diameter coverslips with DF-12 containing 1% fetal calf serum, to allow adhesion. One hour after seeding, coverslips were fixed with 4% paraformaldehyde in 0.1 M phosphate buffer (10 min incubation).

GBM96 cells were seeded on 12 mm diameter coverslips placed in 24-well plates, at a density of 5000 cells/coverslip in GBM medium. Some wells received octanol treatment (1 mM) whereas others received vehicle treatment (ethanol), every 24 h. After 72 h of culture, coverslips were fixed with 4% paraformaldehyde as described above.

Immunohistochemistry for the detection of Cx43 or Panx1 was initiated with a 30 min incubation in a blocking solution containing 2.5% bovine serum albumin in PBS and continued with a 2-h incubation at room temperature in a solution containing the primary antibody (rabbit anti-Cx43 or rabbit anti-Panx1, see Table 1). Then, coverslips were rinsed several times and incubated for 30 min at room temperature with anti-rabbit IgG coupled to FITC (Jackson ImmunoResearch, West Grove, PA, USA, 1:200), prepared in blocking solution. After washing, cells were counterstained with DAPI and mounted on slides as described earlier.

### 4.9. Analysis of Differentiation of Neurosphere-Derived Cells

To analyze differentiation, neurospheres were mechanically dissociated and seeded on poly-D-lysine-treated (Sigma-Aldrich) 12 mm diameter coverslips in DF-12 with 1% fetal calf serum for 4 h to facilitate adhesion, at a density of 10,000 cells/coverslip. Then, coverslips were washed and maintained in DM without growth factor supplementation. Coverslips from the same experiment were divided in two groups: one received drug treatment and the other the corresponding vehicle.

Treatments were performed as follows: octanol was added every 24 h to some cultures from a 5% stock solution prepared in ethanol to reach a final concentration of 0.5 mM whereas control cultures were added with equivalent volumes of ethanol. Gap27 was added from a 100X stock solution prepared in MilliQ^®^ water to reach final concentration of 0.3 mM.

After 48 h of seeding, neurosphere-derived adhered cells in the coverslips were fixed with 4% paraformaldehyde in 0.1 M phosphate buffer (10 min incubation). Immunohistochemistry was always performed in parallel with coverslips from control and treated cultures of the same experiment. Coverslips were incubated for 30 min in a blocking solution containing 2.5% bovine serum albumin in PBS and then in the primary (2 h at room temperature) and, after rinsing, in the secondary (30 min at room temperature) antibodies prepared in blocking solution. After washing, cells were counterstained with 0.1 µg/mL DAPI for 10 min, washed again and mounted on slides with a n-propyl-gallate solution as described before. The primary antibodies used (listed in Table 1) were: doublecortin (DCX, to identify neurons, [57]), glial fibrillary acidic protein (GFAP, to identify astrocytes, [58]), and chondroitin sulphate proteoglycan (NG2, to identify oligodendrocyte precursors, [33]). The secondary antibodies used were anti-mouse IgG labeled with TRITC or with FITC and anti-rabbit IgG labeled with TRITC or with FITC (Jackson ImmunoResearch, West Grove, PA, USA, 1:200).

### 4.10. Epifluorescence Microscopy

Coverslips with neurosphere-derived cells from five to eight independent experiments were visualized via epifluorescence microscopy after the immunohistochemical procedures. Fluorescent images of the cells were captured using a camera DP73 (Olympus, Hamburg, Germany) coupled with an epifluorescence microscope (Olympus BX61, Olympus) with a 20X objective. Omission of primary antibodies resulted in the absence of detectable staining in all cases. Six random fields were captured per coverslip with the excitation wavelengths for the secondary antibody associated fluorophores (FITC or TRITC) and for DAPI.

Counting of BrdU-positive cells and, of apoptotic cells, was performed in the images and expressed as percentage of the total number of cells identified via DAPI staining.

Counting of Cx43-, Panx1-, DCX-, GFAP- or NG2-positive cells was performed on the merged images of the marker with the DAPI channel. Only cells with clear labeling of the cytoplasm or the processes around the DAPI labeled nuclei were considered immunopositive for each marker.

The analysis of the ratios Cx43/DAPI and Panx1/DAPI was performed with the ImageJ software (imagej.net/ij/) (NIH). A custom-written code was used that allowed for the determination of the percentage of area in the image of each marker that was above the user-defined threshold for each marker. The ratio was calculated as the percentage of pixels above the Cx43 or Panx 1 threshold divided by the percentage of pixels above DAPI threshold.

### 4.11. Statistics

Statistics were performed with Sigma Plot 11.0 (Systat Software, San José, CA, USA). All values are expressed as the mean ± standard error of the mean (SEM).

Comparisons between two groups (control and treatment) were achieved by using a Student’s *t* test, for parametric data, and a Mann–Whitney U test for nonparametric data, both at a significant level of *p* < 0.05.

Comparisons between groups in MTT assay experiments with GBM cells were performed with a one-way ANOVA test at a significant level of *p* < 0.05.

## Figures and Tables

**Table 1:** List of antibodies used in the study.

Antibody Name	Immunogen	Antibody Information	Working Concentration
Bromodeoxyuridine (BrdU)	The epitope is inside the DNA helix. DNA must be denatured before antibody efficiently binds to DNA-BrdU	Roche Diagnostics GmBH Mannheim, Germany Cat#11 170 376 001	1:100
Doublecortin (DCX)	Amino acids 123–402 mapping at the C-terminus of doublecortin of human origin	Santa Cruz Biotechnology Santa Cruz, CA, USA Cat# sc-271390 Mouse monoclonal	1:100
Glial Fibrillary Acidic Protein (GFAP)	Purified GFAP from pig spinal cord	Sigma-Aldrich Cat# G3893 Mouse monoclonal	1: 1400
Chondroitin sulphate proteoglycan (NG2)	Purified NG2 chondroitin sulphate proteoglycan from rat	Millipore Cat#AB5320 Rabbit polyclonal	1:400
Connexin43 (Cx43)	Peptide corresponding to a segment of the 3rd cytoplasmic domain (C-terminal portion) of rat connexin43	Thermo Scientific Cat #71-0700 Rabbit polyclonal	1:50
Pannexin1 (Panx1)	Recombinant Protein Epitope Signature Tag (PrEST) antigen sequence available in datasheet	Sigma Aldrich Cat #HPA016930 Rabbit polyclonal	1:500

## Data Availability

Dataset can be provided upon reasonable request.

## Supplementary Materials

The supporting information can be downloaded at: https://www.mdpi.com/article/10.3390/ijms25126288/s1.
