# Supplementary material for: The Gap Junction Inhibitor Octanol Decreases Proliferation and Increases Glial Differentiation of Postnatal Neural Progenitor Cells
Source: Int J Mol Sci. 2024 Jun 7;25(12):6288. doi: 10.3390/ijms25126288 (PMC11203596; doi:10.3390/ijms25126288)
Supplement: Supplementary file 1 [file ijms-25-06288-s001.zip › Figure S1.pdf]

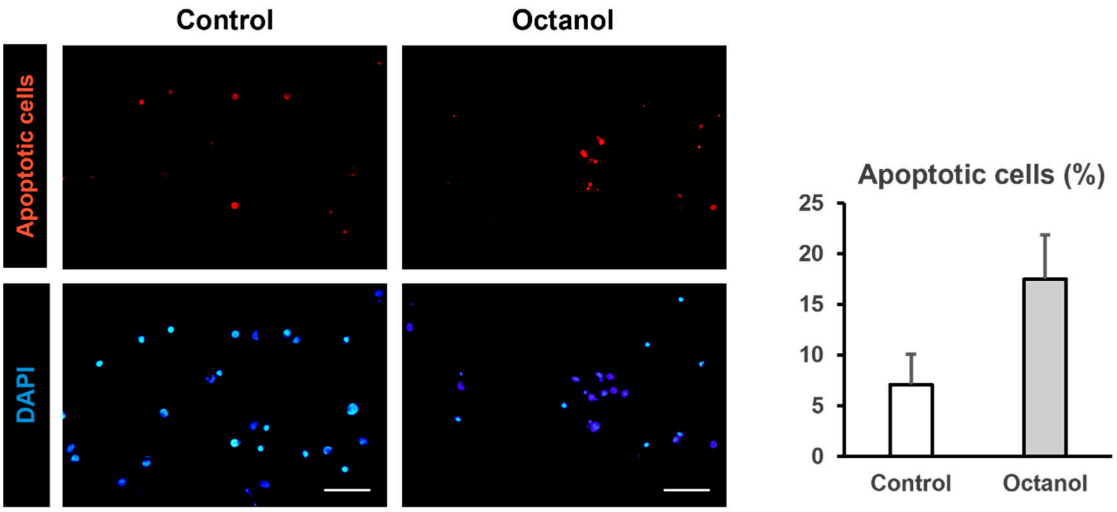

Figure S1. Effect of octanol treatment on neural progenitor cell apoptosis. Neural progenitor cells from the postnatal rat subventricular zone were cultured in the absence (control) or presence of 0.5 mM octanol, and the percentage of apoptotic cells was evaluated by the TUNEL assay after 72 h of culture. Epifluorescence images showing apoptotic cells (in red) in control cultures and in cultures treated with octanol. The total number of cells in each field was identified by DAPI staining (in blue). Bars: 50 μm. The graph shows the percentage of apoptotic cells in each experimental condition. Data are mean ± SEM (n=12 photographs of each experimental condition from 2 independent experiments, $p = 0.061$, Student's t test).
