# Supplementary material for: The Gap Junction Inhibitor Octanol Decreases Proliferation and Increases Glial Differentiation of Postnatal Neural Progenitor Cells
Source: Int J Mol Sci. 2024 Jun 7;25(12):6288. doi: 10.3390/ijms25126288 (PMC11203596; doi:10.3390/ijms25126288)
Supplement: Supplementary file 1 [file ijms-25-06288-s001.zip › Table S1.pdf]

Table S1: Percentage of Panx1-positive cells and Panx1/DAPI ratio in SVZ NPCs and in GBM96 cells in control conditions and after treatment with octanol (0.5 mM for NPCs, 1 mM for GBM96). Data are the mean ± SEM, n = 3–6. No significant differences were found between control and octanol-treated groups (Student's *t* test).

	**Panx1-positive cells (%)**		**Panx1/DAPI ratio**	
	**Control**	**Octanol**	**Control**	**Octanol**
**SVZ NPCs**	91.6 ± 0.2	88.9 ± 0.9	2.3 ± 0.3	1.9 ± 0.03
**GBM96**	61.8 ± 9.4	74.8 ± 10.2	0.6 ± 0.1	0.6 ± 0.1
